# Uncovering the fine print of the CreER^T2^*-LoxP* system while generating a conditional knockout mouse model of *Ssrp1* gene

**DOI:** 10.1371/journal.pone.0199785

**Published:** 2018-06-28

**Authors:** Poorva Sandlesh, Thierry Juang, Alfiya Safina, Michael J. Higgins, Katerina V. Gurova

**Affiliations:** 1 Department of Cell Stress Biology, Roswell Park Cancer Institute, Buffalo, New York, United States of America; 2 Department of Molecular and Cellular Biology, Roswell Park Cancer Institute, Buffalo, New York, United States of America; Cornell University, UNITED STATES

## Abstract

**FA**cilitates **C**hromatin **T**ranscription (**FACT**) is a complex of SSRP1 and SPT16 that is involved in chromatin remodeling during transcription, replication, and DNA repair. FACT has been mostly studied in cell-free or single cell model systems because general FACT knockout (KO) is embryonically lethal (E3.5). FACT levels are limited to the early stages of development and stem cell niches of adult tissues. FACT is upregulated in poorly differentiated aggressive tumors. Importantly, FACT inhibition (RNAi) is lethal for tumors but not normal cells, making FACT a lucrative target for anticancer therapy. To develop a better understanding of FACT function in the context of the mammalian organism under normal physiological conditions and in disease, we aimed to generate a conditional FACT KO mouse model. Because SPT16 stability is dependent on the SSRP1-SPT16 association and the presence of *SSRP1* mRNA, we targeted the *Ssrp1* gene using a CreER^T2^- LoxP approach to generate the FACT KO model. Here, we highlight the limitations of the CreER^T2^-LoxP (Rosa26) system that we encountered during the generation of this model. *In vitro* studies showed an inefficient excision rate of ectopically expressed CreER^T2^ (retroviral CreER^T2^) in fibroblasts with homozygous floxed *Ssrp1*. *In vitro* and *in vivo* studies showed that the excision efficiency could only be increased with germline expression of two alleles of Rosa26CreER^T2^. The expression of one germline Rosa26CreER^T2^ allele led to the incomplete excision of *Ssrp1*. The limited efficiency of the CreER^T2^-LoxP system may be sufficient for studies involving the deletion of genes that interfere with cell growth or viability due to the positive selection of the phenotype. However, it may not be sufficient for studies that involve the deletion of genes supporting growth, or those crucial for development. Although CreER^T2^-LoxP is broadly used, it has limitations that have not been widely discussed. This paper aims to encourage such discussions.

## Introduction

**F**acilitates **C**hromatin **T**ranscription **(FACT)**, a heterodimeric complex of SSRP1 and SPT16, is a histone chaperone that is involved in chromatin remodeling through its binding to histone oligomers and modulation of nucleosome stability in the vicinity of ongoing RNA and DNA synthesis [1]. FACT has been implicated in multiple processes, including transcription, replication, recombination, and repair [1]. Mutations in the FACT subunits can affect the viability of yeast, and general *Ssrp1* knockout (KO) is lethal at 3.5 days post-copulation (dpc) [2]. However, expression of FACT goes down in human and mouse tissues after early embryogenesis. Later, it is detected almost exclusively in the stem cell niches of several organs [3]. FACT is overexpressed in multiple types of aggressive undifferentiated human tumors with poor prognosis [3, 4]. Furthermore, inhibition of FACT (RNAi-mediated) is tolerable for normal cells *in vitro* but causes growth arrest and death of tumor cells, including cancer stem cells [4–7]. These data suggest that targeting FACT presents an opportunity for the development of a novel anticancer therapeutic approach. However, FACT has primarily been studied in single cell models *in vitro* or in cell-free systems [1, 4]. Thus, to understand the role of FACT in post-embryonic development, tumorigenesis, and tumor progression, we attempted to generate an inducible conditional FACT KO mouse model using the site-specific recombinase CreER^T2^. Since both SSRP1 and SPT16 are stable only when bound to each other and RNAi-mediated repression of SSRP1 expression effectively eliminates both subunits [4, 8], we targeted FACT by deleting part of the *Ssrp1* gene.

Bacteriophage-derived Cre is one of the most widely used site-specific recombinases for gene targeting. Cre recognizes a 34 bp nucleotide sequence known as the LoxP site [9]. To achieve conditional KO, the LoxP sequences are inserted into the intronic regions both upstream and downstream of the critical exons to flank/flox (fl) the region of interest of the endogenous gene [10]. Cre recognizes and recombines these sequences resulting in the deletion of a part of the whole gene, which subsequently inactivates gene function [9, 11].

Temporal control of Cre activity can be achieved by using Cre fusion proteins with mutant ligand binding domains of the estrogen receptor (Cre-ER^T^) that can be activated upon tamoxifen binding [9]. The G521R mutant of the ER ligand binding domain (ER^T^) compromises the ER binding of its natural ligand (17-β-estradiol) but promotes the binding of the synthetic ligand tamoxifen or 4-hydroxytamoxifen (4-OHT) [12, 13]. In the absence of tamoxifen, the Cre-ER^T^ fusion protein is located in the cytoplasm and binds to the heat-shock protein Hsp90. In the presence of tamoxifen, Hsp90 is displaced from Cre-ER^T^, allowing the Cre-ER^T^ fusion protein to move into the nucleus by exposing its nuclear localization signal [14–20]. This control over Cre-mediated recombination provides more flexibility for conditional mutagenesis in mice, especially for genes that are essential for early development, such as *Ssrp1*. Cre-ER^T^ has a preference for tamoxifen over estrogen but the selectivity is not sufficient [21]. Therefore, the ER ligand binding domain was further mutated to improve the specificity, resulting in newer versions of Cre-ER^T^ (e.g., MER-Cre-MER and Cre-ER^T2^). Cre-ER^T2^ carries three mutations in the ER ligand binding domain (G400V/M543A/L544A) and has a ten-fold greater sensitivity to 4-OHT compared to Cre-ER^T^ [17]. Since its development, numerous studies have been conducted using Cre-ER^T2^. Despite its popularity, we and others have observed a number of limitations of the Cre-ER^T2^-LoxP system, including low efficiency of recombination and “leakiness” in expression [21–26]. Only a few studies have reported the excision efficiency of this system, which varies from 30 to 80% [21–26]. The low excision rate may potentially be due to the size of the DNA between the two LoxP sites, mosaic expression of CreER^T2^, and incomplete excision of the floxed loci. However, these limitations have not been widely discussed. The low efficiency of excision may be sufficient for studies involving deletion of genes that negatively affect cell growth or viability because of the positive selection of the cells in which recombination has taken place. However, many studies require a higher rate of excision to fully understand the function of a gene (e.g., studies involving the deletion of genes that may have a positive effect on cell growth) because cells with recombination may be easily overgrown by cells without recombination. In our efforts to develop an inducible conditional *Ssrp1* KO mouse model using the CreER^T2^-LoxP system, we encountered multiple problems due to its low efficiency and leakiness. Here, we present these limitations and ways to resolve them.

## Materials and methods

### Reagents and plasmids

Knockout ^™^ DMEM medium (catalog no. 10829018 ThermoFisher) supplemented with 15% fetal bovine serum (FBS) (catalog no.10438026 ThermoFisher) was used for culturing embryonic stem (ES) cells. F-12K medium (Ham’s F-12K nutrient mix Kaighn’s modification medium;, catalog no. 21127022 ThermoFisher) supplemented with 1% Antibiotic-Antimycotic (AA) solution (catalog no. 15240–062 ThermoFisher) and 10% FBS was used for culturing primary fibroblasts. DMEM medium (catalog no. 11995073 GIBCO Life Technologies) supplemented with 10% FBS and 1% penicillin-streptomycin (catalog no. 15140–122 ThermoFisher) was used for culturing immortalized and transformed mouse fibroblasts.

Tamoxifen (catalog no. T5648), 4-OHT (catalog no. H7904), Trichostatin A (catalog no. 58880-19-6), and polybrene (catalog no. H9268) were purchased from Sigma-Aldrich. Collagenase A (catalog no. 10103578001) was purchased from Roche. Hoechst 33342 was purchased from ThermoFisher (catalog no. H3570).

The following antibodies were used for immunoblotting and immunofluorescence: mouse monoclonal anti-p53 (catalog no. PAb421MilliporeSigma), mouse monoclonal anti-SSRP1 (10D1, catalog no. 609702, BioLegend, Inc), mouse monoclonal anti-SPT16 (8D2, catalog no. 607002, BioLegend, Inc), rabbit polyclonal H-RAS C-20 (sc-520, Santa Cruz Biotechnology), rabbit monoclonal anti-Cre recombinase (D7L7L XP^®^, catalog no. 15036, Cell Signaling Technology), and mouse monoclonal anti-β-actin (catalog no. A3854, MilliporeSigma). Horseradish peroxidase-conjugated secondary antibodies were purchased from Santa Cruz Biotechnology. Secondary anti-mouse Alexa Fluor^®^ 594 or 488 (catalog nos. A-11062 and A-11001, Life Technologies) were used for immunofluorescence. pLV-H-Ras^V12^-Bleo or pLV-Bleo lentiviral vectors and pLXS-GSE56Neo (GSE56) and pLXSN retroviral vectors were kindly provided by Dr. Andrei Gudkov (Roswell Park Comprehensive Cancer Center). pMSCV-CreER^T2^puro retroviral plasmid was a gift from Tyler Jacks (Addgene plasmid no. 22776). Gag/Pol retroviral and pLV-CMVΔR packaging plasmids were kindly provided by Dr. Eugene Kandel (Roswell Park Comprehensive Cancer Center).

### Transfection and transduction

Lentiviruses and retroviruses were generated in the 293T cell line (Clontech) transfected with the appropriate expression vector along with packaging plasmids using PolyJet^™^ In Vitro DNA Transfection Reagent (catalog no. SL100688 SignaGen^®^ Laboratories) according to the manufacturer’s instructions. Virus-containing media from 293T cells were collected 48 h and 72 h later, syringe-filtered through a 0.45 μm filter, and transferred to target cells in the presence of polybrene (10 μg/ml).

### Animal housing and maintenance

Animals were maintained at the Roswell Park Comprehensive Cancer Center. All experiments were conducted with approval from the Institute Animal Care and Use Committee at Roswell Park. The facility is certified by the American Association for Accreditation of Laboratory Animal Care in accordance with the current regulations and standards of the U.S. Department of Agriculture and the U.S. Department of Health and Human Services.

### Generation of mutant mice

The *Ssrp1* targeting vector was obtained from the European Mutant Mouse Consortium (EUCOMM) (PG00202_Z_1_D01) to create a conditional knockout of the *Ssrp1* gene. This vector was electroporated into the G4 ES cell line (129:B6 from Andras Nagy’s lab) by the Gene Targeting and Transgenic Shared Resource at Roswell Park Comprehensive Cancer Center. Clones of the cells with an integrated insert were selected using G418. Clones positive for homologous recombination were identified via Southern blotting and then expanded for injection into C57BL/6J albino host embryos. Embryos were transferred into CD-1 pseudopregnant foster mice. Chimeras were generated from three ES cell clones, and germline transmission was achieved.

#### Generation of *Ssrp1*^*fl/fl*^, *Ssrp1*^*fl/+*^, *Ssrp1*^*fl/fl*^CreER^T2+/-^, and *Ssrp1*^*fl/fl*^CreER^T2+/+^ mice

Chimera mice (*Ssrp1*^*Neo LacZ fl/+*^) containing the targeting vector were bred with B6albinoJ (*Ssrp1*^*+/+*^) wild-type mice to generate heterozygous F1 progeny (*Ssrp1*^*Neo LacZ fl/+*^). *Ssrp1*^*Neo LacZ fl/+*^ were crossed with Flpe/FlpO (B6.129s4 Gt (ROSA) 26Sor<tm1 (FLP1) Dym>/J) mice to excise the synthetic cassette (FRT Neo LacZ) to generate heterozygous (*Ssrp1*^*fl/+*^) mice. *Ssrp1*^*fl/+*^ were bred within the same genotype to generate homozygous (*Ssrp1*^*fl/fl*^) mice. *Ssrp1*^*fl/fl*^ were crossed with homozygous CreER^T2^ (B6.129-Gt (ROSA) 26Sor tm1 (CreER^T2^) tyj/J) to generate *Ssrp1*^*fl/+*^ CreER^T2+/-^. *Ssrp1*^*fl/+*^ CreER^T2+/-^ were crossed within the same genotype to generate homozygous mice for both traits (*Ssrp1*^*fl/fl*^ CreER^T2+/+^).

#### ES cell electroporation

ES cell electroporation was performed by the Gene Targeting and Transgenic Shared Resource at Roswell Park Comprehensive Cancer Center. Briefly, SSRP1 mutant clones were grown in complete ES cell medium on a feeder layer in a 100 mm dish. On the day of electroporation, the ES cells were trypsinized to prepare a single cell suspension. A circular plasmid (11 μg) containing a puromycin resistance cassette was electroporated into the cells. Puromycin selection (0.6 μg/mL) was carried out for 3 to 4 days beginning the day after electroporation. Colonies were picked and transferred to individual wells of a 24-well plate one week after electroporation. ES cell clones were tripsinized, and protein and DNA were isolated to assess the excision of FRT sites.

#### Screening of ES clones for homologous recombination using Southern blotting

DNA was isolated from the ES cells using the Wizard ^®^ Genomic DNA Purification Kit (Promega, catalog no. A1120). Southern blotting was performed as previously described [27]. Genomic DNA was digested with either BsrG1 or Ace1/Nde1. DNA was separated on a 0.8% agarose gel, and transferred to a nylon membrane for Southern blotting. The 5’ hybridization probe (included region size: 8821–9200) was generated by PCR using the following primers: forward primer CGCTTGATGCACTTTTGCTA; reverse primer CAGGACGTTGATGGAAGACA (product size: 347). The 3’ hybridization probe (included region size: 28590–30625) was generated by PCR using the following primers: forward primer GCATTGGTTTTCACCCTTGT; reverse primer CCCCACGTGGATAAATTCTG and a product size: 417

#### Genotyping

Genotyping was done using PCR. Tail tips (1 to 2 mm) were dissolved in 200 μL DirectPCR Lysis Reagent (catalog no. 102-T Viagen) containing 0.2 mg/mL Proteinase K (catalog no. AM2546 Ambion) and incubated overnight at 55°C. The crude lysates were incubated at 85°C for 45 min to inactivate Proteinase K and then centrifuged at 10,000 rpm for 1 min to collect the crude DNA sample in the supernatant. The *Ssrp1*^*fl/fl*^, *Ssrp1*^*fl/+*^*and Ssrp1*^*+/+*^ genotypes were determined by PCR using 50 to 100 ng DNA. PCR was carried out using 2x Taq mix (catalog no. FBTAQM, Froggabio) under the following conditions: 95°C 1 min (1 cycle); 35 cycles of 95°C for 30 sec, 61.2°C for 30 sec, and 72°C for 40 sec; 72 °C for 7 min (1 cycle). The following primers were used to identify wild-type (*Ssrp1*^+^) and mutant allele (Ssrp1^NeoLacZfl^): forward primer (P1): TCCTGGAACTCACTCTGTAGACC, reverse primer (P2): TTAAGTGCTAGAGACCTGATGGC and a second reverse primer (P3A): CTTCACTGAGTCTCTGGCATCTC. The PCR products were run on a 2% agarose gel (1x TAE) at 133 V and the bands were observed as following: P1:P2 (~350 bp) for the wild-type allele, P1: P3A (~592) for mutant allele.

To determine the excision of the synthetic cassette via Flp recombination, PCR was performed as follows: 95°C 1 min (1 cycle); 35 cycles of 95°C for 30 sec, 59.6°C for 30 sec, and 72°C for 40 sec; 72 °C for 7 min. (1 cycle) The primers were: forward primer P1: TCCTGGAACTCACTCTGTAGACC and reverse primer P5: AGTTATCTCGACGAAGTTC. The excised band for the synthetic cassette was ~161 bp. The mutant allele without the synthetic cassette (Ssrp1^fl^) was identified P1 and P2 primers (same as above) using the PCR protocol described above. PCR products for wild-type Ssrp1^+^ were observed at ~350bp and for Ssrp1^fl^ were observed at ~533bp. The CreER^T2^ genotype was determined using the Jackson Laboratory genotyping protocol (https://www2.jax.org/protocolsdb/f?p=116:5:0::NO:5:P5_MASTER_PROTOCOL_ID,P5_JRS_CODE:20407,008463). To determine the excision of the critical exons (exons 4 to 9) (Ssrp1^Δ^) via Cre recombination, forward primer P1 was used in conjunction with reverse primer Pcre2 (CTGGTCTGCATTTCACCCTT). The P1:Pcre2 product was approximately 500 bp.

### Generation of primary, immortalized, and transformed cells

For the generation of primary fibroblasts from *Ssrp1*^*fl/fl*^, *Ssrp1*^*+/+*^, *Ssrp1*^*fl/+*^, *Ssrp1*^*fl/fl*^ CreER^T2+/-^, *Ssrp1*^*fl/fl*^CreER^T2+/+^, and *Ssrp1*^*fl*/+^CreER^T2+/+^ mice, mice were anesthetized with isoflurane, and an approximately 2 cm piece of the tail tip was collected. The samples were washed in sterile 1xPBS containing 1% AA. The tail tips were minced into small pieces using a pair of scissors. F-12K medium containing 1% AA and 2mg/ml Collagenase A (10 ml) was added to the samples, which were incubated for 2 h at 37°C with shaking. The samples were centrifuged at 1200 rpm for 10 min at room temperature. The pellet was resuspended in 7 mL F-12K medium containing 10% FBS and 1% AA and placed in a 100 mm plate (catalog no. 40–229690, Krackeler). The plates were kept in a sterile humidified tissue culture incubator at 37°C and 5% CO_2_ for 4 to 5 days to allow cells to adhere to the plate.

To generate immortalized fibroblasts, primary fibroblasts were infected with genetic suppressor element 56 (GSE56) virus and subsequently selected with 500 μg/ml neomycin (G418 Sulfate). Post-selection, the cells were maintained in DMEM medium supplemented with 10% FBS and 1% penicillin-streptomycin.

For the generation of transformed fibroblasts, the immortalized fibroblasts were infected with oncogenic H-RAS^V-12^ virus and selected with bleomycin (30 ug/ml) for approximately one week.

### 1. Excision of *Ssrp1* (in vitro and in vivo)

For the excision of *Ssrp1 in vitro*, primary, immortalized, or transformed cells (1x10^6^) were plated in 150 mm plates. The cells were treated with 2 μM 4-OHT for 96 h (primary cells) or 120 h (immortalized and transformed cells). The medium was replaced every 48 h with fresh 2 μM 4-OHT containing medium. The excision of *Ssrp1* was confirmed at both the DNA and protein level.

For excision of *Ssrp1 in vivo*, *Ssrp1*^*fl/fl*^CreER^T2+/+^ and *Ssrp1*^*fl/fl*^ CreER^T2+/-^ mice were treated with 1mg (100 μL) tamoxifen (stock solution 10 mg/mL in 5% ethanol and 95% corn oil) or 100 μL of vehicle (5% ethanol in corn oil) by intraperitoneal injection for 5 days.

### Immunoblotting

Protein extracts were prepared by lysing cells in Pierce^®^ luciferase cell lysis buffer (2x) (Thermo Scientific, catalog no. 16189) for *in vitro* studies. Protein extracts from mouse spleen were prepared by lysing with 1x RIPA buffer (150 mM sodium chloride, 1% NP-40, 0.5% sodium deoxycholate, 0.1% SDS, 50 mM Tris pH 8.0). Protein concentrations were determined using Quick Start ^™^ Bradford 1x dye reagent (Bio-Rad, catalog no. 500–0205). Equal amounts of protein were run on precast 4 to 20% gradient gels (Bio-Rad, catalog no. 3450028), and transferred to PVDF membranes (Bio-Rad, catalog no.1620177). The membranes were blocked with 5% nonfat milk-PBS buffer and incubated with primary and secondary antibodies according to the manufacturer’s instructions. Western Lightning Plus- ECL (PerkinElmer catalog no. NEL104001) substrate was used to detect the proteins.

### Immunofluorescence staining

Cells were washed with PBS and fixed with 4% paraformaldehyde for 15 min at room temperature. The fixed cells were blocked with PBS containing 3% BSA and 0.2% Triton X-100 for 1 h. The cells were incubated with primary antibody (1:200, anti-SSRP1 or anti-CRE recombinase) in blocking solution for 1 h. The samples were washed three times in wash solution (6x dilution of blocking solution in PBS) for 10 min each and then incubated with secondary anti-mouse Alexa Fluor^®^ antibody (1:500) in washing solution for 45 min. The cells were washed three times in washing solution for 10 min each and then counterstained with Hoechst 33342 (1 μg/mL) for 10 min. Images were acquired using a Zeiss Axio Observer A1 inverted microscope with N-Achroplan 100x/1.25 oil lens, Zeiss MRC5 camera, and AxioVision Rel.4.8 software. Image analysis and quantification were performed using ImageJ software. Each image was divided into four quadrants, and ten cells in each quadrant were analyzed to determine the corrected total cell fluorescence (CTCF) using the following equation: CTCF = integrated density–(area of the selected cell x the mean fluorescence of background readings).

### Methylene blue staining

Primary (1x10^6^) or immortalized or transformed (5x10^5^) fibroblasts were seeded in duplicate in a 6-well plate and allowed to grow to confluency (~ 3 to 4 days). Cells were fixed and stained with 0.5% methylene blue staining solution (1 ml per well) in 50% methanol (, catalog no. M4159 MilliporeSigma) for 10 min at room temperature with shaking. The stained cells were washed with water, and the plates were air dried.

### Quantitative PCR (qPCR)

Genomic DNA was prepared from cells and tail tissue using the Wizard^®^ Genomic DNA Purification Kit (, catalog no. A1120 Promega) and DirectPCR Lysis Reagent (catalog no. 102-T Viagen), respectively. The following primers were used: GAPDH Forward (AACTTTGGCATTGTGGAAGG), GAPDH Reverse (ACACATTGGGGGTAGGAACA), F1 forward (GGAACCGAAGTTCCTATTCCG), R2 reverse (GCCCTCAGCTGAGTACTCAA), and R3 reverse (ACACCGCCTACTGCGACTAT). Mutant allele expression was determined using F1 and R3 (F1:F3). Expression of the excised gene was determined using F1 and R2 (F1:R2). qPCR was performed using Applied Biosystems Power SYBR^®^ Green PCR Master Mix (, catalog no. 4367659 ThermoFisher) according to the manufacturer’s instructions using the default parameters of the 7900HT sequence detection system (ABI PRISM; Applied Biosystems).

To compare gene expression between samples, the threshold cycle (CT) value was normalized using the mean CT for the reference gene (GAPDH) to obtain the ΔCT. To quantify the excised allele (F1:R2) and mutant allele (F1:R3) in 4-OHT/tamoxifen-treated samples, the ΔΔCT was calculated using the equation ΔCT F1:R2 - ΔCT F1:R3. The final data were expressed as the fold difference defined as 2^**-(ΔCT F1:R2 - ΔCT F1:R3)**^.

### β-Galactosidase (*lacZ*) activity assay

Lungs, testis, colon, and pancreas were harvested from *Ssrp1*^*+/+*^ and *Ssrp1* ^*LacZ Neo fl* /+^ mice and washed in 1x PBS on ice. Tissues were fixed (1x PBS+ 0.2% glutaraldehyde + 2% formaldehyde) on ice for 45 min. Tissues were washed three times (15 min each) in wash buffer (2 mM MgCl_2_, 0.01% sodium deoxycholate, and 0.02% nonidet P-40 in 1x PBS) and then incubated with staining solution (1mg/ml X-gal (in dimethylformaldehyde), 5 mM potassium ferricyanide, 5 mM potassium ferrocyanide, 2 mM MgCl_2_, 0.01% sodium deoxycholate and 0.02% nonidet P-40 in 1x PBS) for 3 h at 37°C in the dark. After staining, Tissues were washed three times with 1x PBS for 15 min each at room temperature.

### Statistical analysis

The unpaired t-test (Mann-Whitney test) was used for comparisons of quantitative data from the control and treated groups. Analyses were conducted using GraphPad Prism 7.03. All p-values are two-sided.

## Results

### I. Generation of a mouse model with conditional deletion of *Ssrp1 (Ssrp1*^*fl/fl*^*)*

**Fig 1:**
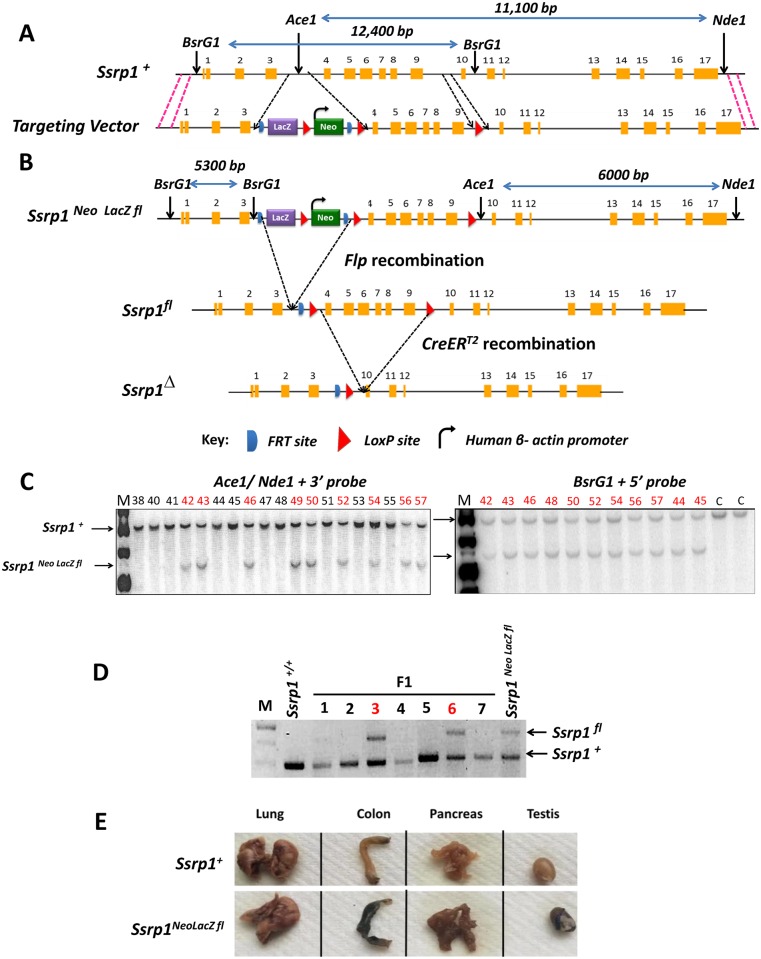
Generation of conditional *Ssrp1* KO mice. A) Schematic representation of the wild-type *Ssrp1* allele (*Ssrp1*^+^) and the targeting vector with 5’ and 3’ homology arms (pink dashed lines). The black dashed lines indicate intron regions in *Ssrp1*^+^ where LoxP sites, FRT sites, and the synthetic cassette are inserted. B) Schematic representation of the mutant allele (*Ssrp1* ^*Neo LacZ fl*^) after homologous recombination, representation of mutant allele without the synthetic cassette (*Ssrp1*^*fl*^) after Flp-FRT recombination and representation of *Ssrp1*^*Δ*^ allele with deletion of the critical exons after CreER^T2^-LoxP recombination. C) Southern blot hybridization of DNA from ESC cells to determine clones with correct (red) and incorrect (black) recombination of 3’ and 5’ arm. Wild-type DNA used as control for 5’ probe is C. D) PCR of genomic DNA from F1 progeny of wild-type (*Ssrp1*^*+/+*^) crossed with chimera (*Ssrp1* ^*Neo LacZ fl /*+^). Two bands are present in heterozygous pups (*Ssrp1* ^*Neo LacZ fl* /+^) (red). Numbers indicate individual pup IDs in one litter. E) β-Galactosidase assay to determine LacZ activity (Blue-Green stain for positive activity) on SSRP1 positive (colon, pancreas and testis) and negative (lung) tissues isolated from *Ssrp1*^+/+^ and *Ssrp1* ^*Neo LacZ fl* /+^ mice.

Previously, we showed that FACT stability is dependent on the dimerization of its two subunits (i.e., SPT16 and SSRP1) [8]. Furthermore, SPT16 is only stable when SSRP1 mRNA is present in the complex [8]. Therefore, a targeting vector that allowed for conditional KO of *Ssrp1* was used to generate a conditional FACT KO mouse model. The EUCOMM targeting vector consisted of the complete *Ssrp1* gene with two LoxP sites flanking exons 4 to 9 of *Ssrp1* and a synthetic cassette. The synthetic cassette consisted of a promoter-less *LacZ* gene and neomycin-resistance gene (*Neo*^*r*^) under the control of the β-actin promoter. A third LoxP site was located upstream of the β-actin promoter. This synthetic cassette, which was flanked by two FRT sites, was within the intronic region between exons 3 and 4 of *Ssrp1* (Fig 1A). Similar to the Cre-LoxP system, the Flp-FRT site-specific recombinase system uses FRT sites that are recognized and recombined by Flp recombinase [28]. The targeting vector was electroporated into G4 (B6.129s) mouse ES cells. Following selection with G418, Southern blot-based screening for the 3’ and 5’ recombination arms was used to identify clones containing the homologous recombination of the mutant (*Ssrp1*^*Neo LacZ fl*^) *Ssrp1* allele in place of the endogenous wild-type allele (*Ssrp1*^+^) (Fig 1C). Chimeras were generated and subsequently bred with wild-type mice (*Ssrp1*^*+/+*^) to obtain an F1 generation of heterozygous (*Ssrp1*^*Neo LacZ fl/+*^) mice. Their genotypes were determined by PCR (Fig 1D).

*Ssrp1* is not expressed ubiquitously [3] in adult mice. *LacZ* did not have its own promoter and could be expressed under the control of the endogenous *Ssrp1* promoter. β-galactosidase staining was performed to compare the expression of mutant and wild-type alleles. Positive staining was observed in tissues from heterozygous mice that expressed SSRP1, including testes (high FACT expression), colon (moderate FACT expression), and pancreas (weak FACT expression). Staining was absent in tissues that do not express SSRP1 (e.g., lung). Thus, the *Ssrp1*^*Neo Lacz fl*^ allele had a similar expression pattern to that of the wild-type *Ssrp1*^+^ allele. Tissues from wild-type mice were negative for LacZ staining (Fig 1E).

For future studies, we wanted to use LacZ as a reporter of mutant *Ssrp1* expression. Thus, we generated mice expressing the *LacZ* gene but lacking the expression of the *Neo*^*r*^ gene (*Ssrp1* ^*LacZ fl/ LacZ fl*^). To generate *Ssrp1* ^*LacZ fl/ LacZ fl*^ mice, *Ssrp1* ^*Neo LacZ fl* /+^ mice were bred to *Ssrp1* ^*Neo LacZ fl*/+^ mice to generate homozygous mice (*Ssrp1* ^*Neo LacZ fl*/ *Neo LacZ fl*^). *Ssrp1* ^*Neo LacZ fl*/ *Neo LacZ fl*^ mice carried three LoxP sites. When crossed with CreER^T2^ mice, three different genotypes were possible: 1) *Ssrp1* ^*LacZ fl/ LacZ fl*^ in which Neo^r^ was excised, leaving the critical exons flanked by the LoxP sites; 2) *Ssrp1* ^*LacZ Δ /LacZ Δ*^ in which both Neo^r^ and critical exons were excised; 3) *Ssrp1*^*Neo LacZ Δ / Neo LacZ Δ*^ in which critical exons were excised, but the synthetic cassette remained intact.

Breeding of *Ssrp1* ^*Neo LacZ fl* /+^ to *Ssrp1* ^*Neo LacZ fl* /+^ resulted in 59% *Ssrp1* ^*Neo LacZ fl* /+^ and 40.9% *Ssrp1* ^+/+^ pups based on 22 pups from three litters. However, homozygous pups (*Ssrp1* ^*Neo LacZ fl/ Neo LacZ fl*^) were not obtained. Because the expression of at least one *Ssrp1* allele is necessary to maintain embryonic viability at early stages of embryonic development [2], we hypothesized that the lack of generation of *Ssrp1* ^*Neo LacZ fl/ Neo LacZ fl*^ mice might be due to the lack of SSRP1 expression from the mutant allele.

**Fig 2:**
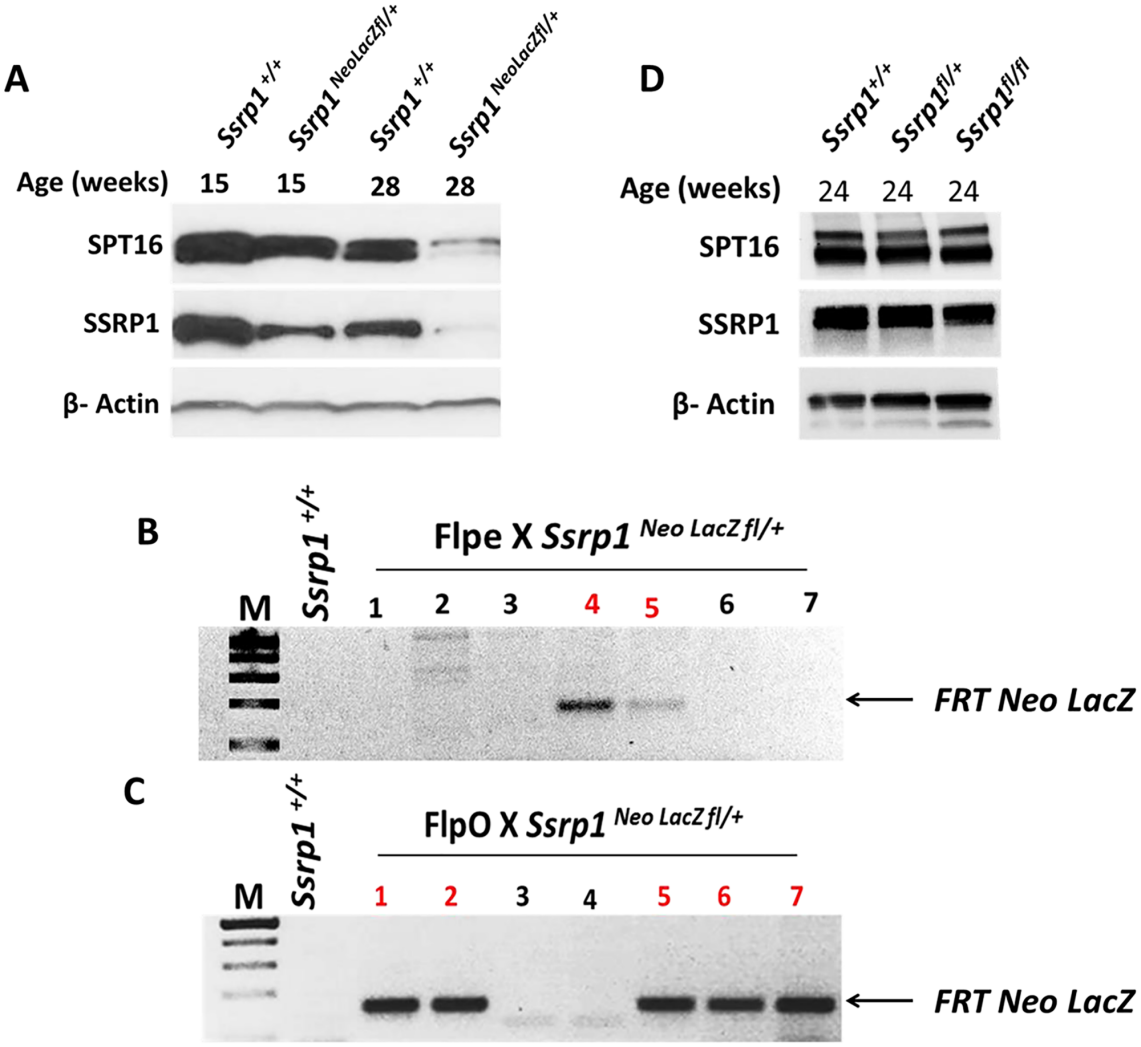
Generation of homozygous floxed Ssrp1 mice (*Ssrp1* ^*fl/fl*^). A) Immunoblot of protein extracts from spleen illustrating the difference in SSRP1 and SPT16 levels between *Ssrp1*^*+/+*^ and *Ssrp1* ^*Neo LacZ fl/+*^ age-matched mice (15 weeks and 28 weeks of age). B & C) Representative PCR of genomic DNA from individual pups (indicated by numbers) from a single litter of B) *Ssrp1*^*Neo LacZ fl/+*^ crossed with B6.129S ROSA26 FLPe (Flpe) and C) *Ssrp1*^*Neo LacZ fl/+*^ crossed with B6.129S ROSA26 FLPo (FlpO) mice to confirm pups with excision of the synthetic cassette (FRT Neo LacZ) shown in red. D) Immunoblot of protein extracts from spleen showing SSRP1 and SPT16 expression in *Ssrp1*^*+/+*^, *Ssrp1*^*fl/+*^, and *Ssrp1*^*fl/fl*^ mice.

To test this hypothesis, we compared the SSRP1 and SPT16 protein levels in the spleens (a FACT-positive organ in adult mice) of *Ssrp1*^+/+^ and *Ssrp1* ^*Neo LacZ fl* /+^ mice from two different pairs of littermates (15 weeks and 28 weeks of age). The results showed that the expression of both subunits was approximately two times higher in the *Ssrp1*^+/+^ mice compared to the *Ssrp1* ^*Neo LacZ fl* /+^ mice. These data suggested the absence of expression from the *Ssrp1* ^*Neo LacZ fl*^ allele, and could explain the absence of homozygous *Ssrp1* ^*Neo LacZ fl*^ pups (Fig 2A). We hypothesized that the strong β-actin promoter used for *Neo* expression might be interfering with *Ssrp1* expression. However, we later learned that the targeting vector provided by the EUCOMM was predicted to generate a null allele through splicing to the lacZ trapping element contained in the synthetic cassette. Unfortunately, this characteristic of the synthetic cassette was not explicitly explained by EUCOMM and has only been discussed in one publication [29].

To generate homozygous floxed *Ssrp1* mice, we had to remove the synthetic cassette using Flp-mediated FRT recombination. *Ssrp1* ^*Neo LacZ fl* /+^ mice were crossed with mice expressing Flp recombinase (Flpe). From this cross, only ~28% of the progeny had the synthetic cassette excised based on 14 pups from two litters (Fig 2B), which suggested that the rate of excision at the FRT sites by Flpe was low. To overcome this problem, we obtained mice expressing FlpO recombinase. According to the literature, FlpO has a higher recombination efficiency compared to Flpe [30, 31]. Use of the FlpO recombinase resulted in ~43% of the progeny undergoing recombination based on 14 pups from two separate litters (Fig 2C).

To determine if the excision efficiency could be improved *in vitro*, ES cells were electroporated with either Flpe or FlpO plasmid and tested for the excision of the synthetic cassette using PCR (S1 Fig). Similar to the *in vivo* data, after selection of the ES cells for the expression of Flpe or FlpO, only 22% and 50% of the clones, respectively, showed excision of the synthetic cassette. Thus, mosaic excision of the synthetic cassette from the mutant allele was observed both *in vitro* and *in vivo*.

FRT-excised *Ssrp1*^*fl/+*^ mice were bred with each other (*Ssrp1*^*fl/+*^ x *Ssrp1*^*fl/+*^), resulting in progeny with either *Ssrp1*^*fl/fl*^, *Ssrp1*^*fl/+*^, or *Ssrp1*^+/+^genotypes. The protein levels of SSRP1 and SPT16 in the spleen of the heterozygous mice (*Ssrp1*^*fl/+*^) were now comparable to that of *Ssrp1*^*+/+*^ mice (Fig 2D).

### II. Excision of *Ssrp1* with ectopically expressed CreER^T2^ *in vitro*

**Fig 3:**
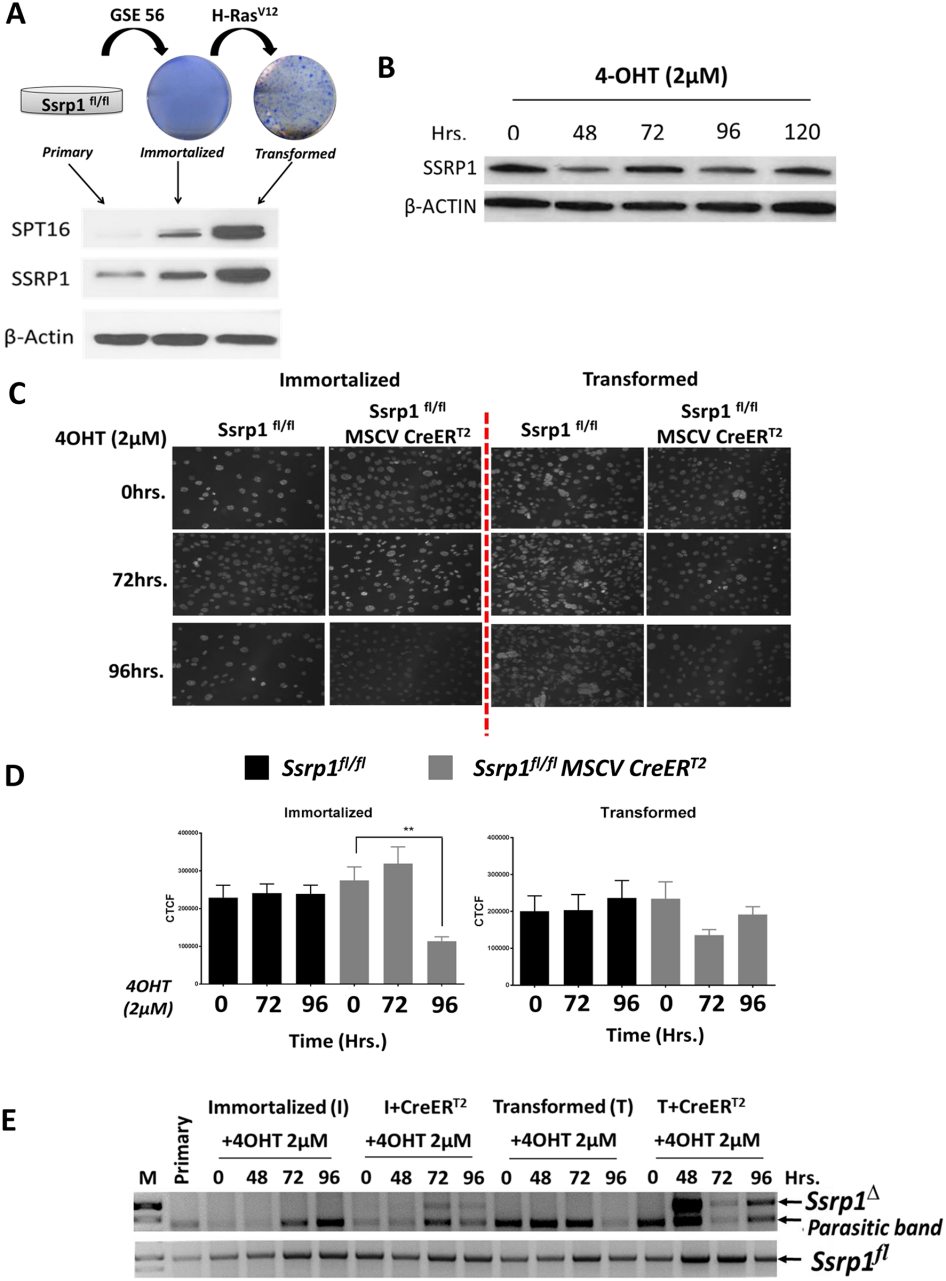
Excision efficiency of ectopically expressed CreER^T2^ (MSCV CreER^T2^). A) Methylene Blue staining of immortalized and transformed *Ssrp1*^*fl/fl*^ fibroblasts and immunoblot determining the levels of SSRP1 and SPT16 in primary, immortalized and transformed cells. B) Immunoblot to determine time-dependent changes in SSRP1 protein levels in immortalized Ssrp1^fl/fl^ MSCV CreER^T2^ upon treatment with 2 μM 4-OHT. C) SSRP1 Immunofluorescence in immortalized and transformed *Ssrp1* ^*fl/fl*^ +/- MSCV CreER^T2^ before or after treatment with 2 μM 4-OHT for 72 or 96 h. D) Quantification of SSRP1 immunofluorescence staining to calculate the Corrected Total Cell Fluorescence (CTCF). E) PCR of the genomic DNA to determine the excision of *Ssrp1* after 4-OHT treatment of immortalized and transformed *Ssrp1*^*fl/fl*^ fibroblasts with or without transduction with MSCV CreER^T2^.

While generating mice for *in vivo* experiments, we tested the excision of the *Ssrp1*^*fl*^ allele *in vitro* using fibroblasts isolated from the tails of *Ssrp1*^*+/+*^, *Ssrp1*^*fl/+*^, and *Ssrp1*^*fl/fl*^ mice. We previously observed very low FACT levels in primary non-tumor cells. However, FACT levels were increased upon oncogene-induced transformation of the primary cells [32]. Therefore, to establish *in vitro* FACT-positive cells, we immortalized primary tail fibroblasts using the dominant negative p53 **g**enetic **s**uppressor **e**lement 56 (GSE56 [32]) and then transformed the immortalized cells using H-Ras^V12^ oncogene. As expected, immortalized and transformed cells expressed higher levels of the FACT subunits (Fig 3A). Immortalized and transformed cells were infected with retroviral CreER^T2^ recombinase (MSCV CreER^T2^with puromycin selection marker [33]) followed by puromycin selection. After selection, cells were treated with 4-OHT (2 μM), an active metabolite of tamoxifen. SSRP1 protein levels were slightly reduced at some time points after the start of treatment with 4-OHT (48 to 120 h), but no clear time-dependence was observed (Fig 3B, 3C and 3D). PCR analysis confirmed excision of the critical exons of *Ssrp1* (*Ssrp1*^*Δ*^) following treatment with 4-OHT. However, the presence of an unrecombined *Ssrp1*^*fl*^ allele was also detected (Fig 3E). These data suggested that the excision was happening only in a fraction of cells in the population or only one *Ssrp1*^*fl*^ allele was excised. These results may be due, in part, to the heterogeneity among cells in the population with respect to Cre-recombinase expression levels.

**Fig 4:**
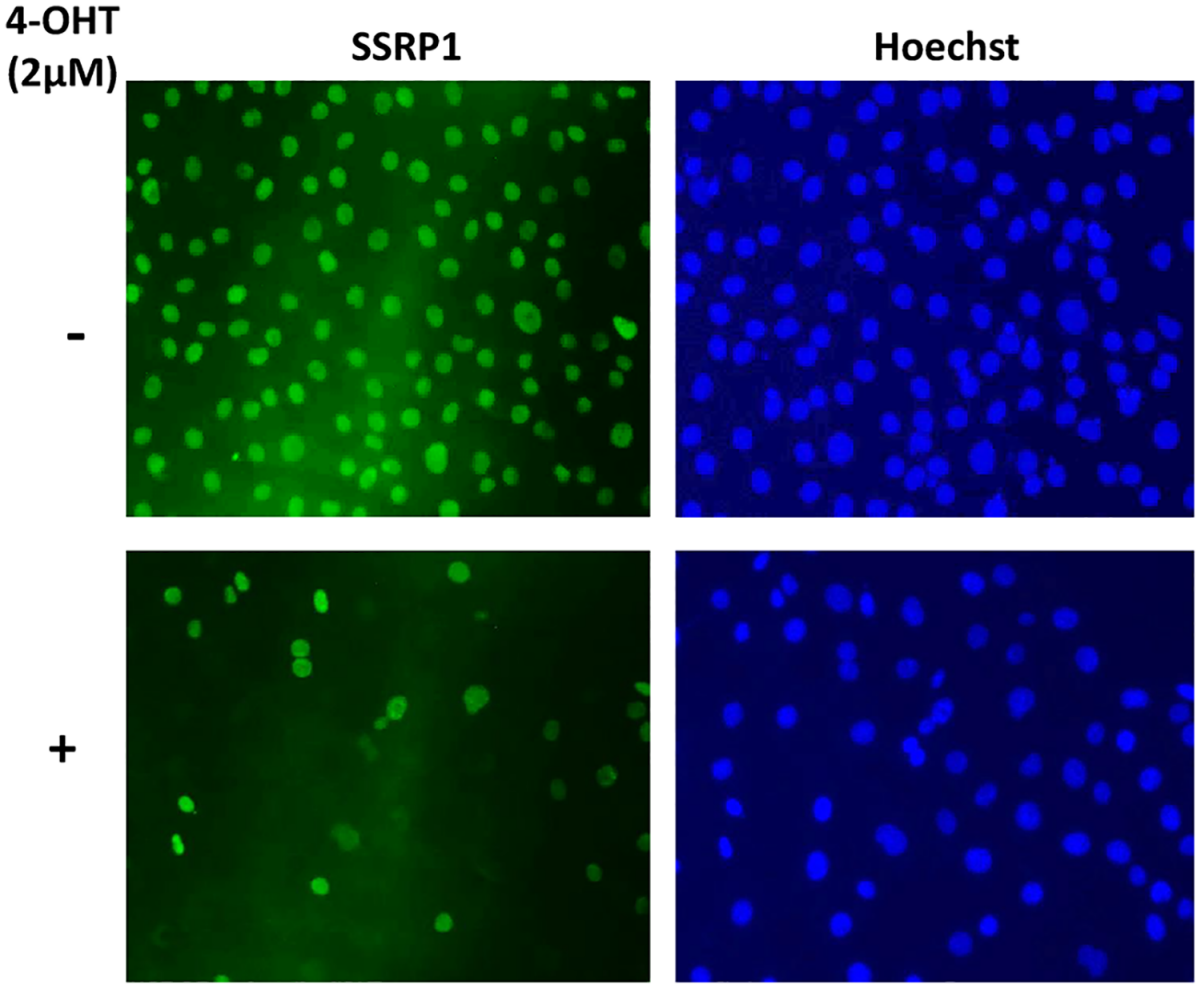
Mosaic excision of Ssrp1 in a clone generated by limiting dilution. An example of a clone developing from a single transformed *Ssrp1*^*fl/fl*^ MSCV CreER^T2^ cell. SSRP1 immunofluorescence (green) in the presence and absence of treatment with 2 μM 4-OHT for 120 h. DNA was counterstained with Hoechst.

To test this hypothesis, single cell clones of transformed *Ssrp1*^*fl/fl*^ MSCV CreER^T2^ fibroblasts were generated by limiting dilution. Cloned cells were subjected to 4-OHT treatment, and SSRP1 immunofluorescence was performed to identify the clones with complete excision of *Ssrp1*. Variable excision was observed within each clone, and no clones demonstrated uniform excision in all cells in response to 4-OHT (Fig 4 and S2 Fig). Thus, excision did not occur in all cells and, moreover, excision of both alleles occurred in almost none of the cells.

**Fig 5:**
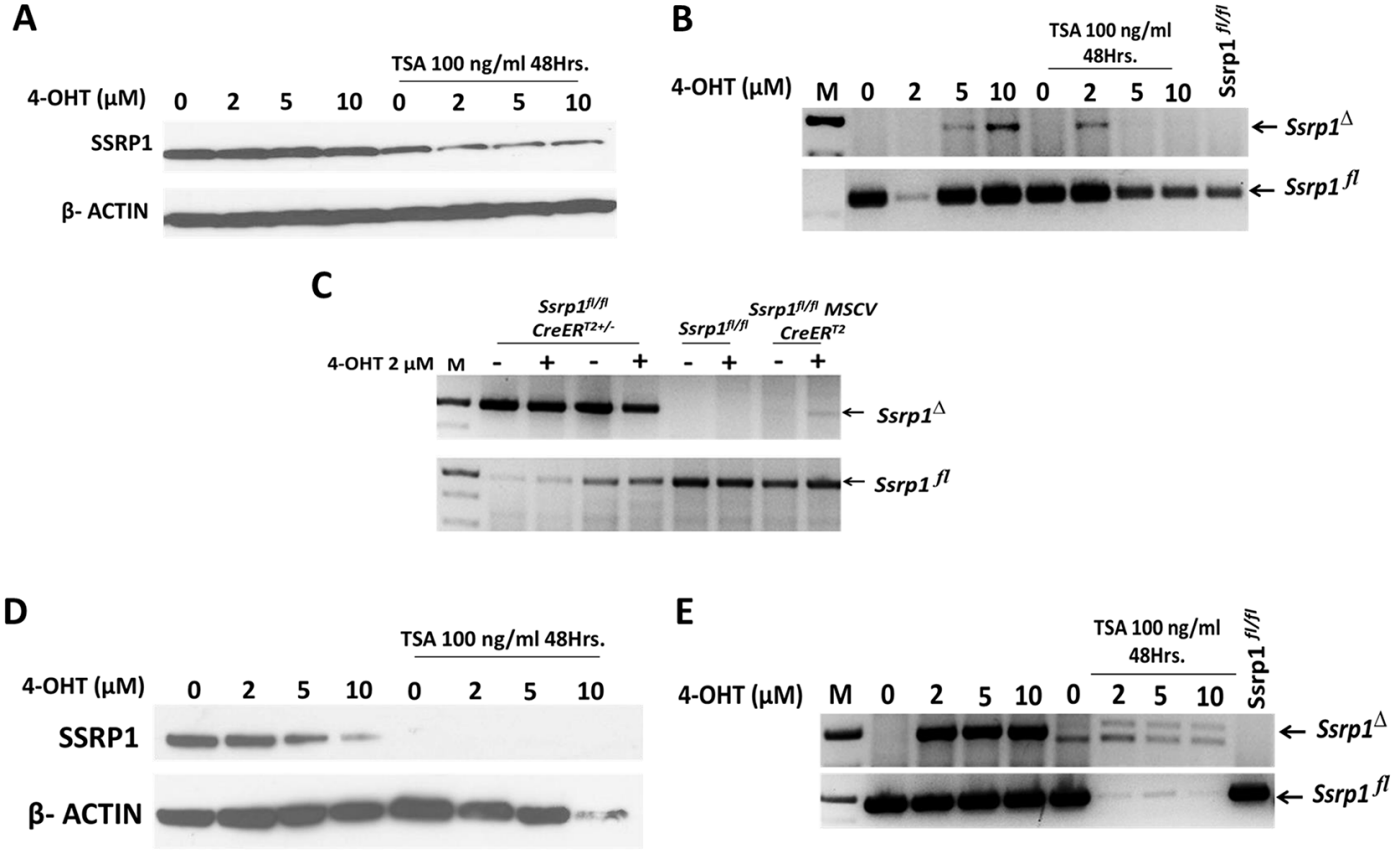
Effect of TSA on *Ssrp1*^*fl*^ excision by ectopic and germline CreER^T2^. A) Immunoblot of SSRP1 protein expression in transformed *Ssrp1*^*fl/fl*^ MSCV CreER^T2^ cells with or without 4-OHT and TSA treatment. B) PCR of genomic DNA of transformed *Ssrp1*^*fl/fl*^ MSCV CreER^T2^ cells with or without 4-OHT and TSA treatment. C) PCR of genomic DNA of immortalized *Ssrp1*^*fl/fl*^ *CreER*^*T2*+/-^ with or without 2 μM 4-OHT treatment for 120 h. D) Immunoblot of SSRP1 protein expression in immortalized *Ssrp1*^*fl/fl*^ *CreER*^*T2*+/-^ cells with or without 4-OHT and TSA treatment. E) PCR of genomic DNA of immortalized *Ssrp1*^*fl/fl*^ *CreER*^*T2*+/-^ cells with or without 4-OHT and TSA treatment.

This low recombination efficiency could be due to the inaccessibility of the LoxP sites to the Cre recombinase. To test this hypothesis, we assessed the excision efficiency of CreER^T2^ in cells with a more open chromatin state achieved using treatment with trichostatin A (TSA), an inhibitor of histone deacetylase (HDAC). Immortalized *Ssrp1*^*fl/fl*^ MSCV CreER^T2^ fibroblasts were treated with a range of 4-OHT concentrations in the absence or presence of TSA (100 ng/ml) for 48 h. SSRP1 protein levels were reduced following 4-OHT treatment in the presence of TSA. However, a reduction in SSRP1 protein levels was also observed with TSA alone (Fig 5A). Genomic DNA PCR showed no improvement in the excision efficiency, suggesting that the opening of chromatin was not sufficient to improve the recombination efficiency of ectopically expressed CreER^T2^ (Fig 5B).

Another possibility for the low excision efficiency was low Cre-ER^T2^ expression. To test this hypothesis, we evaluated the cytoplasmic and nuclear distribution of CreER^T2^ protein with and without 4-OHT treatment. Immunofluorescence staining for Cre and SSRP1 in transformed *Ssrp1*^*fl/fl*^ fibroblasts transduced with MSCV CreER^T2^ was performed following 4-OHT treatment. In the absence of 4-OHT, cytoplasmic Cre was observed in a small fraction (<1%) of cells (S3 Fig). Treatment of cells with 4-OHT resulted in an increased number of Cre-positive cells, but never 100% of the cells. Importantly, Cre was localized primarily in the nucleus of all positive cells in the presence of 4-OHT (S3 Fig). These results suggested that CreER^T2^ was unstable in the absence of 4-OHT. Although all the cells in the population were resistant to puromycin, some had suppressed CreER^T2^ expression.

### III. Excision of Ssrp1 in cells from mice with germline expression of CreER^T2^

The results presented thus far suggested that ectopic expression of CreER^T2^ did not lead to efficient homozygous excision of *Ssrp1* in *Ssrp1*^*fl/fl*^ fibroblasts. Hence, we determined whether germline-expressed CreER^T2^ was able to excise *Ssrp1* effectively. *Ssrp1*^*fl/fl*^ mice were crossed with homozygous Rosa26 CreER^T2^ (CreER^T2+/+^) mice. Since the literature suggests that even one allele of CreER^T2^ is sufficient for the excision of a gene of interest [34–36], heterozygous mice for both alleles (*Ssrp1*^*fl*/+^ CreER^T2+/-^) were bred to get mice homozygous for floxed-*Ssrp1* with at least one allele of CreER^T2^ (*Ssrp1*^*fl/fl*^CreER^T2+/-^). Fibroblasts were isolated from tail tissue of *Ssrp1*^*fl/fl*^CreER^T2+/-^ mice and then immortalized and transformed. To test the efficiency of germline CreER^T2^, immortalized *Ssrp1*^*fl/fl*^ CreER^T2+/-^ cells were treated with 4-OHT for 120 h. *Ssrp1* excision in these cells was assessed using PCR (Fig 5C). The results showed the presence of a recombined *Ssrp1* allele in both 4-OHT-treated and untreated samples, which suggested that germline CreER^T2^ is leaky. Moreover, the *Ssrp1*^*fl*^ allele was still observed in 4-OHT-treated cells, which suggested that like retroviral CreER^T2^, germline CreER^T2+/-^ may not be active in all cells. Alternatively, there was only a low chance of excising both alleles together or cells with both alleles excised do not survive. However, no toxicity was observed upon treatment with 4-OHT.

To determine if the excision efficiency of germline CreER^T2+/-^ could be improved when only one allele was excised, we treated immortalized *Ssrp1*^*fl/fl*^CreER^T2+/-^ fibroblasts with 4-OHT (round 1) for 120 h. Genomic DNA was isolated from both treated and untreated cells to assess the excision of the *Ssrp1* allele. At the same time, the treated cells were re-plated and subjected to a second round of 4-OHT treatment for 120 h. Consistent with the previous results, a single round of 4-OHT treatment resulted in incomplete excision as indicated by the presence of both *Ssrp1*^*fl*^ and *Ssrp1*^*Δ*^ bands on Part A in S4 Fig. Moreover, the second round of treatment did not lead to excision of both alleles. These results suggest that the inefficiency of a single copy of germline-expressed CreER^T2^ may not be because of its inability to excise two alleles but may be due to inaccessibility of the LoxP sites to the Cre recombinase. To test this hypothesis, we treated cells with 4-OHT in the presence or absence of TSA in an attempt to make the chromatin more accessible., The efficiency of the single germline copy of CreER^T2^ following treatment with 4-OHT and TSA increased compared to the ectopically expressed CreER^T2^ as seen by the decrease in the *Ssrp1*^*fl*^ band in genomic DNA analysis (Fig 5E). In addition, the absence of SSRP1 protein upon TSA treatment showed that the opening of the chromatin enhanced the excision rate of endogenously expressed CreER^T2^. However, SSRP1 was absent following TSA treatment even in the absence of 4-OHT. These data suggest that either CreER^T2+/-^ is leaky or the inhibition of HDAC causes a reduction of FACT subunit protein levels or both (Fig 5D).

**Fig 6:**
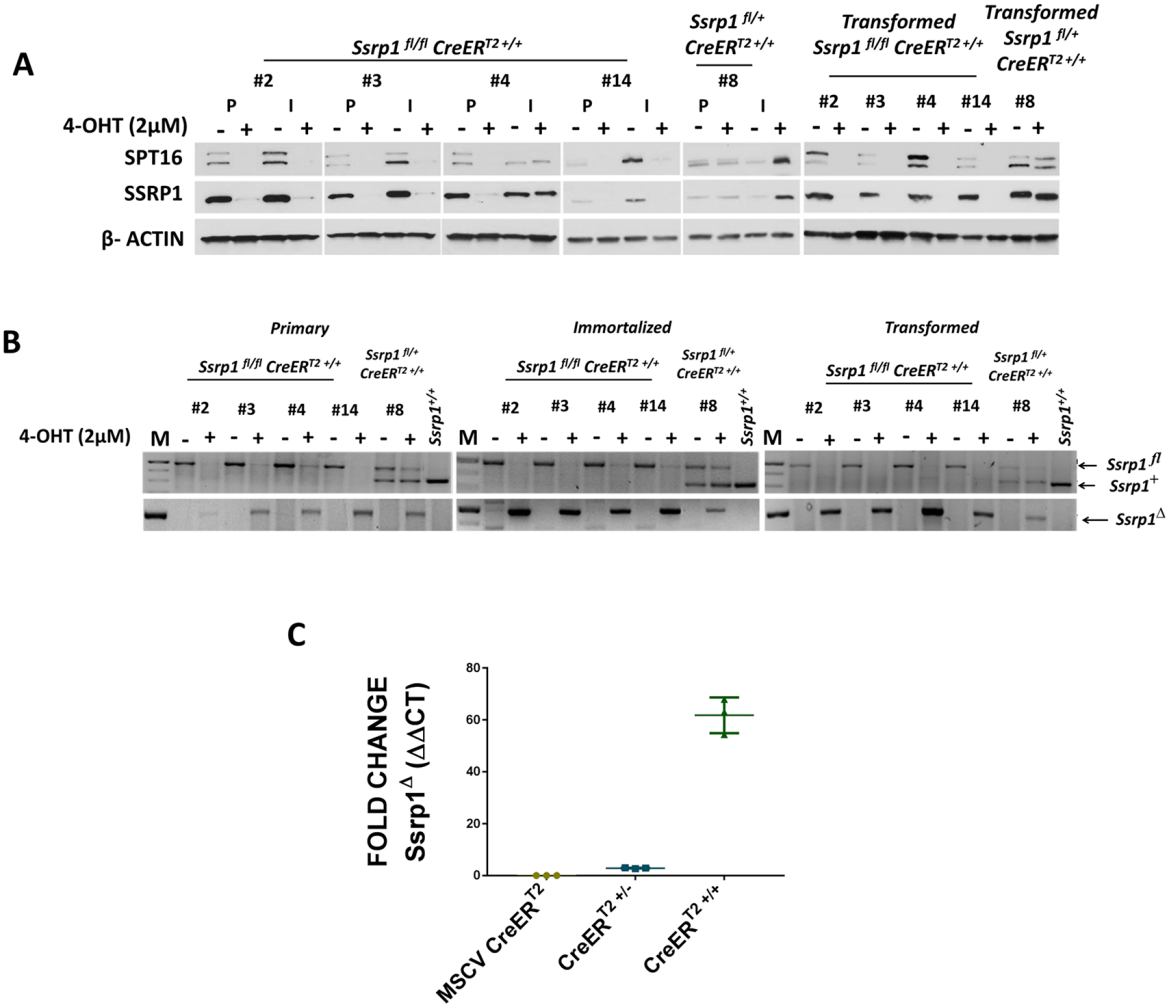
Excision efficiency of *Ssrp1* ^*fl/fl*^ by two alleles of germline-expressed *CreER*^*T2*^. A) Immunoblot to determine SSRP1 and SPT16 protein expression in primary, immortalized, and transformed *Ssrp1*^*fl/fl*^ *CreER*^*T2*+/+^ and *Ssrp1* ^*fl/+*^ *CreER*^*T2*+/+^ fibroblasts with or without 4-OHT treatment (2 μM) for 120 h. B) PCR of genomic DNA to confirm excision of Ssrp1 in primary, immortalized, and transformed *Ssrp1*^*fl/fl*^ *CreER*^*T2*+/+^ and *Ssrp1* ^*fl/+*^ *CreER*^*T2*+/+^ fibroblasts with or without 4-OHT treatment (2 μM) for 120 h. C) qPCR to determine the relative expression of the excised gene in *Ssrp1*^*fl/fl*^ MSCV CreER^T2^, *Ssrp1*^*fl/fl*^ *CreER*^*T2*+/-^ and *Ssrp1*^*fl/fl*^ *CreER*^*T2*+/+^ cells when treated with 2 μM 4-OHT for 120 h.

Since the CRE-mediated recombination efficiency was low when only one allele of CreER^T2^ was present, we tested whether the presence of two alleles of CreER^T2^ would be more efficient. We generated mice homozygous for both floxed *Ssrp1* and CreER^T2^ (*Ssrp1*^*fl/fl*^CreER^T2+/+^), and then generated primary, immortalized, and transformed cells as described above. 4-OHT treatment significantly reduced SSRP1 levels in all cell types (Fig 6A). PCR analysis of genomic DNA also confirmed excision of the critical exons of Ssrp1 and absence of the mutant allele of SSRP1 upon treatment with 4-OHT (Fig 6B). Thus, expression of CreER^T2^ from two alleles resulted in a much more efficient recombination, which in turn was more efficient than recombination from ectopically expressed CreER^T2^ (MSCV CreER^T2^) (Fig 6C).

### IV. Excision of *Ssrp1* in mice with germline CreER^T2^

**Fig 7:**
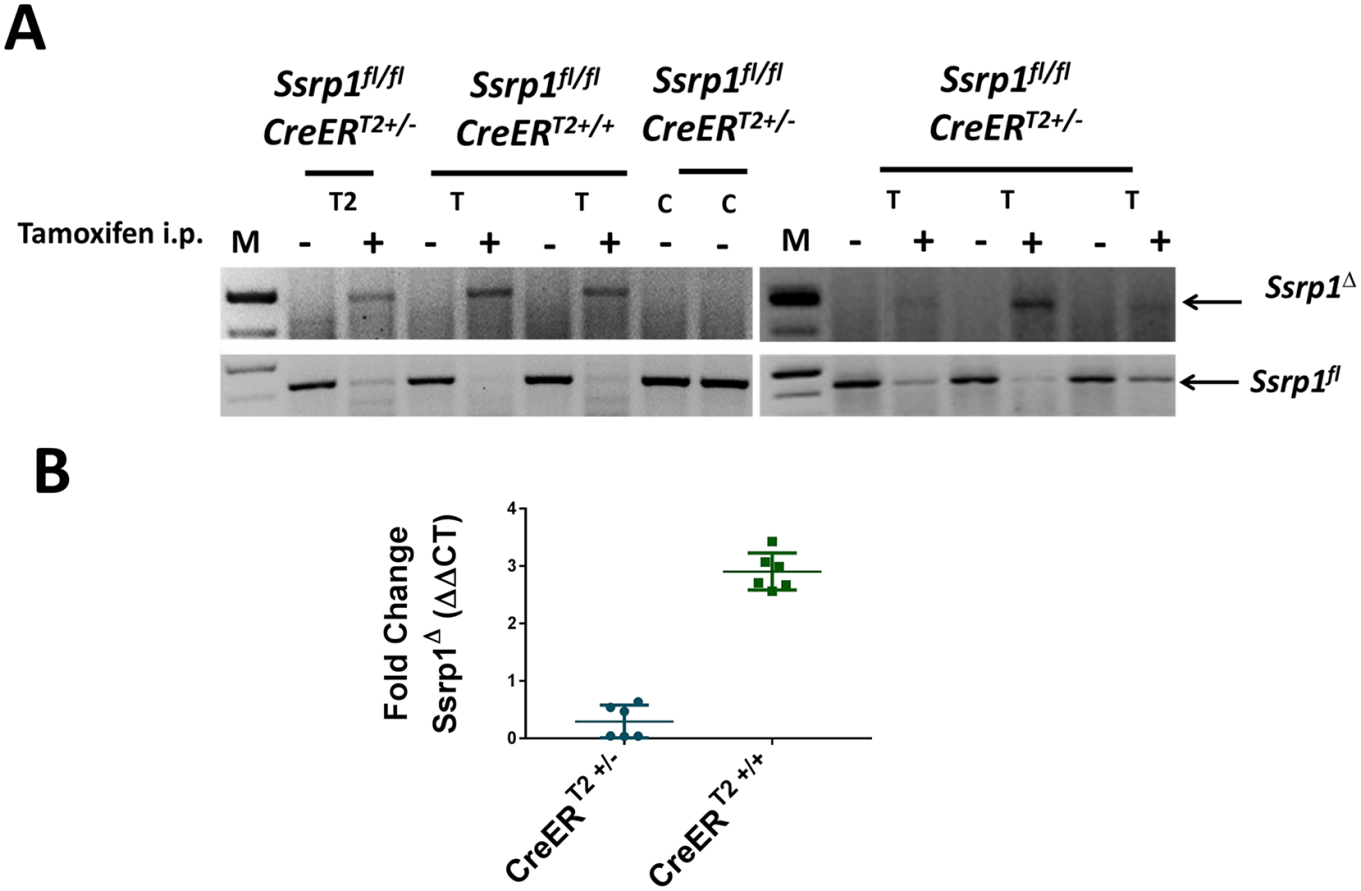
Excision efficiency of two alleles of germline *CreER*^*T2*^ in vivo. A) PCR of genomic DNA to determine *Ssrp1* excision in *Ssrp1*^*fl/fl*^ *CreER*^*T2*+/-^ and *Ssrp1*^*fl/fl*^ *CreER*^*T2*+/+^ mice treated with 1 mg tamoxifen or *Ssrp1*^*fl/fl*^*CreER*^*T2*+/-^ mice treated with vehicle control i.p. for 5 days. T = tamoxifen-treated mice and C = vehicle-treated control mice; T2 = *Ssrp1*^*fl/fl*^ *CreER*^*T2*+/^ mouse treated twice with tamoxifen. For T2, the image shows the PCR results following the second round of treatment.^-^B) qPCR to determine the relative expression of the excised gene in *Ssrp1*^*fl/fl*^ *CreER*^*T2*+/-^ and *Ssrp1*^*fl/fl*^ *CreER*^*T2*+/+^ mice treated with 1 mg tamoxifen i.p. for 5 days. p-value = 0.0022.

To compare the efficiency of *Ssrp1*^*fl*^ excision by one or two copies of germline CreER^T2^ *in vivo*, we treated *Ssrp1*^*fl/fl*^CreER^T2+/+^ and *Ssrp1*^*fl/fl*^CreER^T2+/-^ mice with 1 mg/day tamoxifen and two *Ssrp1*^*fl/fl*^CreER^T2+/-^ mice with vehicle control intraperitoneally for five days. DNA was collected from the mice seven days after the final treatment to determine the excision efficiency. PCR analysis showed complete excision of the *Ssrp1*^*fl*^ gene in the *Ssrp1*^*fl/fl*^CreER^T2+/+^ mice (Fig 7A). One of the *Ssrp1*^*fl/fl*^CreER^T2+/-^ mice was treated twice with tamoxifen to mimic the *in vitro* study. However, two rounds of treatment did not result in complete excision but there was a slight decrease in the intensity of the *Ssrp1*^*fl*^ band after the second round of treatment (Fig 7A and Part B in S4 Fig). SSRP1 protein levels correlated with the excision of the gene (S5 Fig). qPCR of genomic DNA from these mice showed that there was a 2.5-fold higher rate of Ssrp1 excision in *Ssrp1*^*fl/fl*^CreER^T2+/+^ mice compared to *Ssrp1*^*fl/fl*^CreER^T2+/-^ mice (Fig 7B). Because of the small sample size, the differences did not reach statistical significance. However, these observations potentially indicate that, like *in vitro*, two alleles of Rosa26 CreER^T2^ are required to achieve complete excision of *Ssrp1* in mice.

## Discussion

CreER^T2^-mediated gene inactivation is a powerful tool to study a gene of interest with temporal and spatial control. However, to fully understand the function of a gene, it is important that the Cre recombination be efficient and complete. Here, we report the limitations of the CreER^T2^ system encountered while generating the *Ssrp1* KO mouse model. The results of this study highlight the inefficiencies of this system and provide insights on how to increase the recombination efficiency. We understand that each case of genetic engineering potentially has specific limitations, which may result from specific gene or genomic features. Thus, the experience that we report here may only be fully applicable to *Ssrp1*. Nevertheless, we believe it is pertinent to disclose such observations to develop a better understanding of the most widely used system for genetic manipulation.

The excision efficiency of CreER^T2^ is very low when expressed ectopically through retroviral transduction. Differential excision efficiency of each floxed allele was seen after treatment with 4-OHT, which may have been due to: 1) mosaic expression of CreER^T2^ (i.e., not all cells express CreER^T2^) even though they were puromycin resistant and incomplete excision of the gene occurs [37]. It is possible that CreER^T2^ was active in the absence of any ligand or was activated by estrogen or other steroids present in the FBS, which might lead to excision of Ssrp1 in untreated cells (i.e., negative selection for the cells with active CreER^T2^ in the absence of tamoxifen). 2) CreER^T2^ might not be in an active state in the cells. In particular, clonal variation in the excision of *Ssrp1* indicated the loss of CreER^T2^ activity in certain cells generated from a single cell clone. Analysis of the cytoplasmic and nuclear levels of CreER^T2^ suggested that this protein was not stable in the absence of its ligand as only a few cells stained positive for CreER^T2^ in the cytoplasm in the absence of 4-OHT even though they were puromycin resistant. However, the number of cells with positive nuclear stain increased in the presence of 4-OHT. These results are not unprecedented as many steroid receptors (e.g., androgen receptor) are unstable in the absence of their ligand [38]. Thus, due to the lack of CreER^T2^ activity, partial excision of *Ssrp1* might occur. 3) Ectopically expressed CreER^T2^ might not be able to access the floxed gene due to a closed chromatin state. Upon treatment with an HDAC inhibitor, the chromatin might become more accessible, leading to complete excision by CreER^T2^. However, our results suggest that this explanation is less likely because complete excision did not occur even in the open chromatin state caused by TSA (Fig 5A and 5B).

Expression of a single germline copy of Rosa 26 CreER^T2^ (CreER^T2+/-^) also resulted in incomplete excision of Ssrp1. We found that CreER^T2+/-^ was leaky (*in vitro*) as both *Ssrp1*^*Δ*^ and *Ssrp1*^*fl*^ were present in the absence of 4-OHT treatment (Fig 5C). Although partial excision of *Ssrp1* using ectopically expressed CreER^T2^ was not due to inaccessibility of Cre to the chromatin, this was not true for the single germline copy of Cre. Treatment of *Ssrp1*^*fl/fl*^CreER^T2+/-^ immortalized cells with TSA and 4-OHT increased the degree of excision of *Ssrp1* (Fig 5D and 5E). We also saw the presence of *Ssrp1*^*Δ*^ in cells treated with TSA alone (i.e., no 4-OHT treatment), confirming that CreER^T2+/-^ was leaky. FACT expression is critical for the survival of transformed cells and, to some extent, immortalized cells [4]. Therefore, cells expressing CreER^T2^ might have undergone complete excision of *Ssrp1* in the absence of 4-OHT but did not survive. Only cells that had incomplete excision of *Ssrp1* survived. Thus, we could see both *Ssrp1*^*Δ*^ and *Ssrp1*^*fl*^ bands in our assay and the presence of SSRP1 in these cells. Therefore, expression of a single copy of Rosa 26 CreER^T2^ allele was inefficient in yielding homozygous excision of *Ssrp1*. However, we found that two copies of Rosa26 CreER^T2^ expressed in the germline led to complete excision of Ssrp1 both *in vitro* and in *in vivo*. Hence, to achieve homozygous excision of *Ssrp1*, two endogenously expressed alleles of Rosa26 CreER^T2^ are required. Furthermore, determination of excision frequency is necessary for correct assessment of results.

The CreER^T2^-LoxP system was developed in the late 1990s to early 2000s. It has led to many novel and interesting discoveries, and this system prevails even today in the field of gene targeting. With the development of CRISPR technology, the use of Cre-LoxP has been reduced. However, the use of CreER^T2^-LoxP remains constant as a frequently used recombinase system to generate a KO mouse model with temporal and spatial control. Our study describes important factors to consider when employing this system to generate a conditional KO mouse model, particularly of a gene that may play a necessary role in tumor promotion. Our results may not be applicable to studies involving inducible KO of a tumor suppressor gene or the knock-in of a gene.

## Supporting information

S1 Fig Excision of synthetic cassette mediated by Flp recombinases in ES cells. PCR of genomic DNA extracted from ES clones positive for Ssrp1^Neo LacZ fl/+^, and electroporated with A) Flpe recombinase or B) FlpO recombinase to confirm the excision of the synthetic cassette (FRT Neo LacZ).
(PDF)

S2 Fig Mosaic excision of Ssrp1 in a clone generated from a single cell. Examples of single cell colonies of transformed Ssrp1^fl/fl^ MSCV CreER^T2^. SSRP1 immunofluorescence in the presence or absence of 4-OHT (2 μM) treatment for 120 h.
(PDF)

S3 Fig Expression of Cre in presence or absence of ligand. Cre immunofluorescence (Green), in transformed Ssrp1^fl/fl^ transduced with MSCV CreER^T2^ with and without 4-OHT treatment. DNA was counterstained with Hoechst 33342. Arrows (white) indicate cells with Cre in the cytoplasm in the absence of 4-OHT.
(PDF)

S4 Fig Excision efficiency of *Ssrp1* ^*fl/fl*^ by single allele of germline-expressed *CreER*^*T2*^. A) PCR of genomic DNA to determine the excision of *Ssrp1* ^*fl*^ *in Ssrp1*^*fl/fl*^ *CreER*^*T2*+/-^ immortalized fibroblasts. Cells were treated with 2 μM 4-OHT for 120 h for round 1 of treatment. The cells were re-plated for a second round of treatment with 2 μM 4-OHT for 120 h. B) PCR of genomic DNA to determine excision of *Ssrp1* in *Ssrp1*^*fl/fl*^ *CreER*^*T2*+/-^ mice following one round of treatment with 1 mg/day tamoxifen or control vehicle i.p. for five days.
(PDF)

S5 Fig Changes in SSRP1 and SPT16 protein levels upon excision of *Ssrp1* by germline *CreER*^*T2*^ *in vivo*. Immunoblot of protein extracts from mouse spleen to determine SSRP1 and SPT16 protein expression in mice treated with 1 mg/day tamoxifen or vehicle i.p. for five days. T = tamoxifen-treated mice and C = vehicle-treated mice; T2 = *Ssrp1*^*fl/fl*^ *CreER*^*T2*+/^ mouse treated twice with tamoxifen.
(PDF)
