# Supplementary figures and images for: Uncovering the fine print of the CreERT2-LoxP system while generating a conditional knockout mouse model of Ssrp1 gene
Source: PLoS One. 2018 Jun 28;13(6):e0199785. doi: 10.1371/journal.pone.0199785 (PMC6023160; doi:10.1371/journal.pone.0199785)

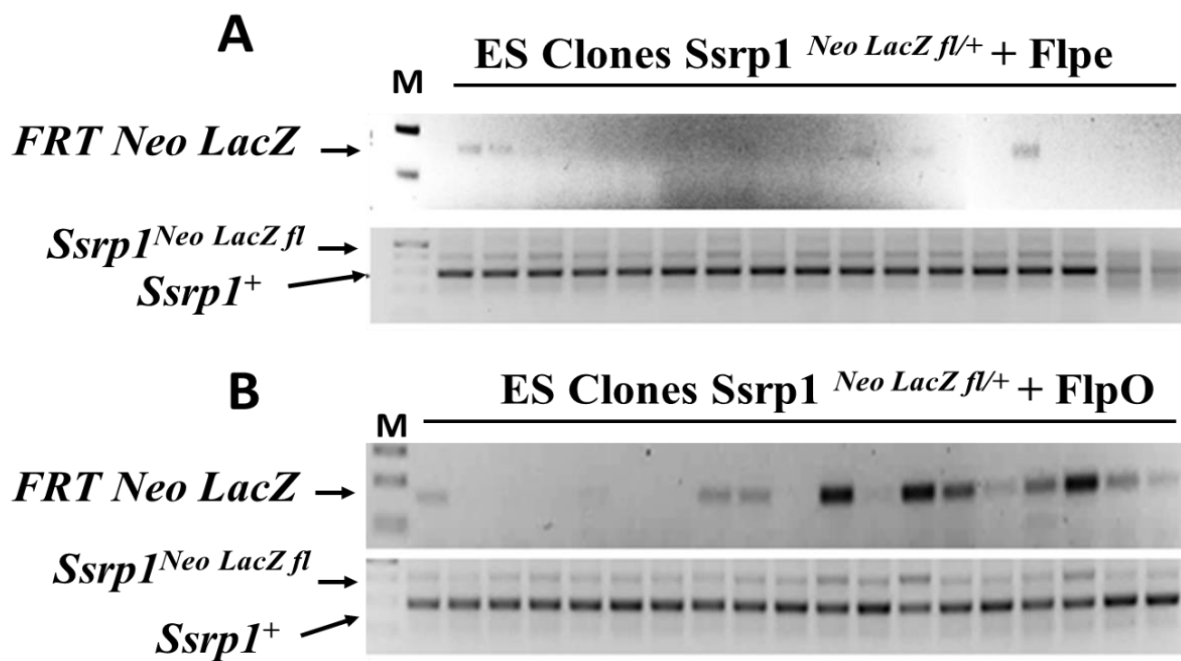

S1 Fig

Supplement: S1 Fig — PCR of genomic DNA extracted from ES clones positive for Ssrp1Neo LacZ fl/+, and electroporated with A) Flpe recombinase or B) FlpO recombinase to confirm the excision of the synthetic cassette (FRT Neo LacZ). (PDF) [file pone.0199785.s001.pdf]

4OHT (2 $\mu$ M)

-

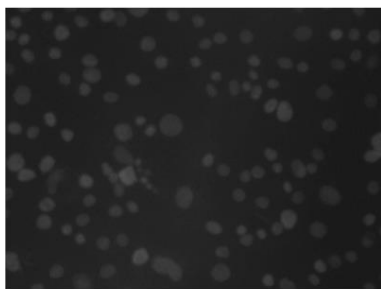

+

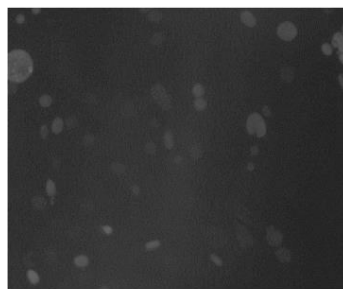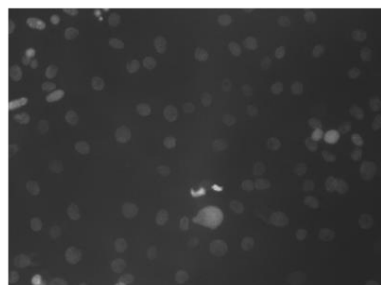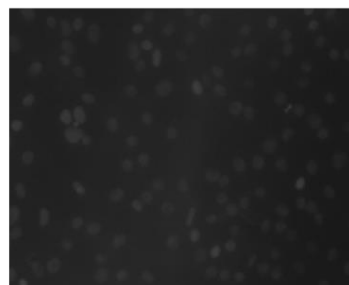

S2 Fig

Supplement: S2 Fig — Examples of single cell colonies of transformed Ssrp1fl/fl MSCV CreERT2. SSRP1 immunofluorescence in the presence or absence of 4-OHT (2 μM) treatment for 120 h. (PDF) [file pone.0199785.s002.pdf]

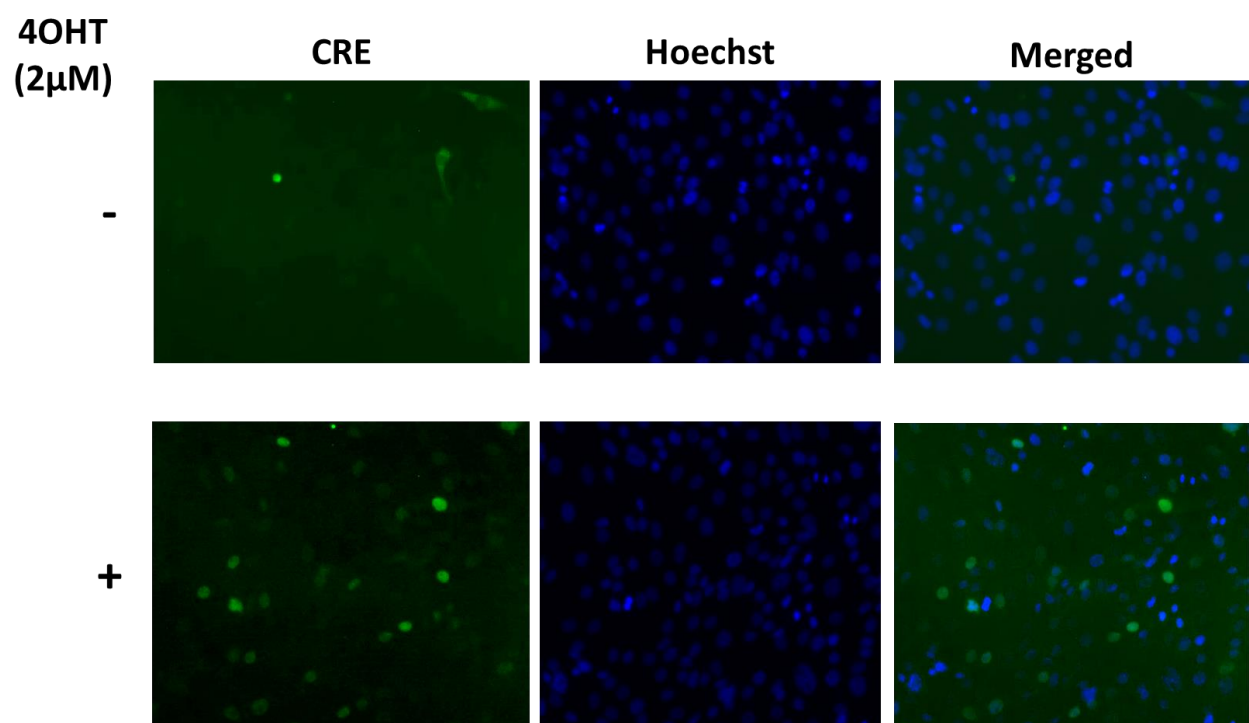

S3 Fig

Supplement: S3 Fig — Cre immunofluorescence (Green), in transformed Ssrp1fl/fl transduced with MSCV CreERT2 with and without 4-OHT treatment. DNA was counterstained with Hoechst 33342. Arrows (white) indicate cells with Cre in the cytoplasm in the absence of 4-OHT. (PDF) [file pone.0199785.s003.pdf]

**A**

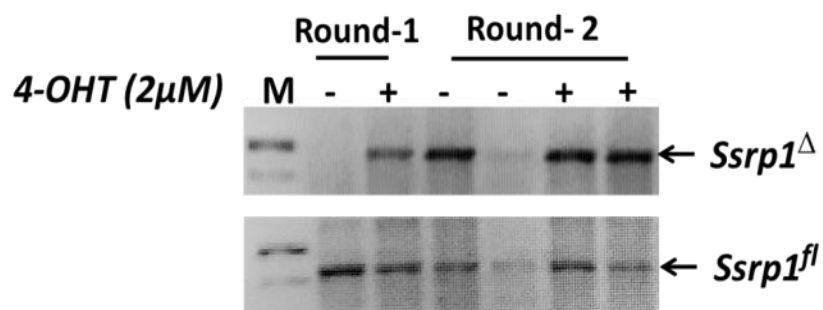

**B**

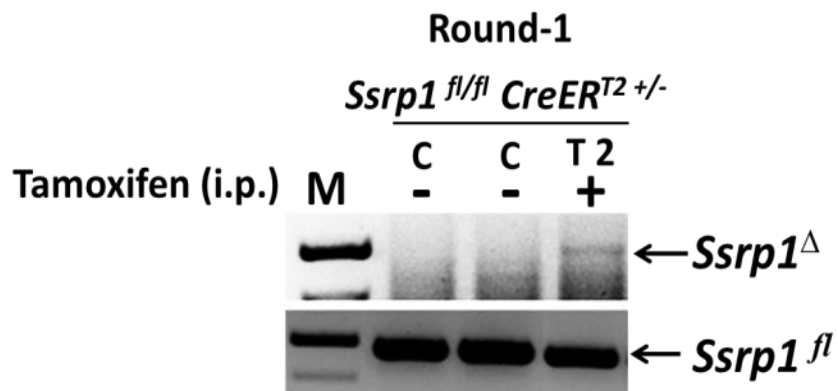

S4 Fig

Supplement: S4 Fig — A) PCR of genomic DNA to determine the excision of Ssrp1 fl in Ssrp1fl/fl CreERT2+/- immortalized fibroblasts. Cells were treated with 2 μM 4-OHT for 120 h for round 1 of treatment. The cells were re-plated for a second round of treatment with 2 μM 4-OHT for 120 h. B) PCR of genomic DNA to determine excision of Ssrp1 in Ssrp1fl/fl CreERT2+/- mice following one round of treatment with 1 mg/day tamoxifen or control vehicle i.p. for five days. (PDF) [file pone.0199785.s004.pdf]

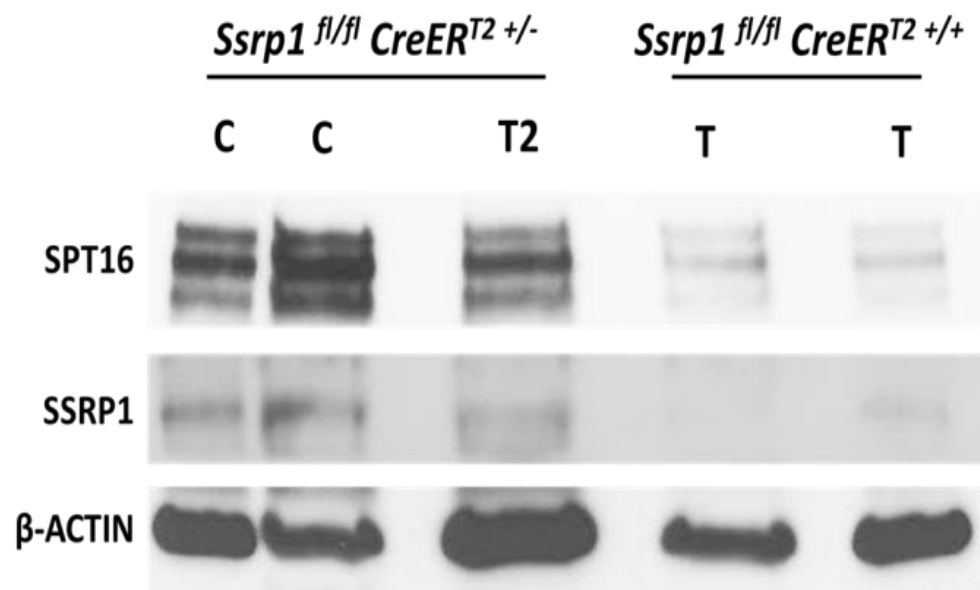

S5 Fig

Supplement: S5 Fig — Immunoblot of protein extracts from mouse spleen to determine SSRP1 and SPT16 protein expression in mice treated with 1 mg/day tamoxifen or vehicle i.p. for five days. T = tamoxifen-treated mice and C = vehicle-treated mice; T2 = Ssrp1fl/fl CreERT2+/ mouse treated twice with tamoxifen. (PDF) [file pone.0199785.s005.pdf]
